# Salvinorin A reduces mechanical allodynia and spinal neuronal hyperexcitability induced by peripheral formalin injection

**DOI:** 10.1186/1744-8069-8-60

**Published:** 2012-08-23

**Authors:** Francesca Guida, Livio Luongo, Gabriella Aviello, Enza Palazzo, Maria De Chiaro, Luisa Gatta, Serena Boccella, Ida Marabese, Jordan K Zjawiony, Raffaele Capasso, Angelo A Izzo, Vito de Novellis, Sabatino Maione

**Affiliations:** 1 Department of Experimental Medicine, Section of Pharmacology, The Second University of Naples, Naples, Italy; 2 Department of Experimental Pharmacology, University of Naples Federico II, Naples, Italy; 3 Department of Pharmacognosy, School of Pharmacy, University of Mississippi, University, MS, USA

**Keywords:** Salvinorin A, Formalin injection, Allodynia, Nociceptive spinal neurons, Glial cells

## Abstract

**Background:**

Salvinorin A (SA), the main active component of *Salvia Divinorum,* is a non-nitrogenous kappa opioid receptor (KOR) agonist. It has been shown to reduce acute pain and to exert potent antinflammatory effects. This study assesses the effects and the mode of action of SA on formalin-induced persistent pain in mice. Specifically, the SA effects on long-term behavioural dysfuctions and changes in neuronal activity occurring at spinal level, after single peripheral formalin injection, have been investigated. Moreover, the involvement of microglial and glial cells in formalin-induced chronic pain condition and in SA-mediated effects has been evaluated.

**Results:**

Formalin induced a significant decrease of mechanical withdrawal threshold at the injected and contralateral paw as well as an increase in the duration and frequency, and a rapid decrease in the onset of evoked activity of the nociceptive neurons 7 days after formalin injection. SA daily treatment significantly reduced mechanical allodynia in KOR and cannabinoid receptor 1 (CB1R) sensitive manner. SA treatment also normalized the spinal evoked activity*.* SA significantly reduced the formalin-mediated microglia and astrocytes activation and modulated pro and anti-inflammatory mediators in the spinal cord.

**Conclusion:**

SA is effective in reducing formalin-induced mechanical allodynia and spinal neuronal hyperactivity. Our findings suggest that SA reduces glial activation and contributes in the establishment of dysfunctions associated with chronic pain with mechanisms involving KOR and CB1R. SA may provide a new lead compound for developing anti-allodynic agents via KOR and CB1R activation.

## Background

Abnormal pain responses such as mechanical allodynia and thermal hyperalgesia are commonly associated with chronic pain states. Following peripheral or central nervous system (CNS) insults, neuron sensitization occurs leading to pain transmission facilitation [1,2]. Spinal glia (microglia and astrocytes) strongly contribute to the development and maintenance of chronic pain [3,4]. In pathological condition, microglia cells undergo to activation resulting in morphological changes and in the release of several neuromodulators, contributing to the mechanisms of central sensitization [5-7]. Once activated, they recruit other microglia and extend the inflammation by activating astrocytes, which are required for the maintenance of the pain state [8]. The involvement of spinal glial and microglial cells in pain regulation is supported by the use of specific metabolic inhibitors [9,10] and their activation has been described in several animal pain models, including peripheral nerve injury [11-13] and intra-plantar zymosan or formalin administration [14-16]. Formalin injection into the hind-paw of rodents is a pain model widely used to investigate the mechanisms of persistent nociception. It produces a severe and immediate peripheral inflammation and nocifensive behaviour, but it is also associated with the development of allodynia tactile and spinal microglial morphological changes lasting at least 14 days [17]. The opioid system is strongly implicated in pain control mechanisms [18-21]. Opioid μ, κ and δ receptors (MOR, KOR, DOR) activation at peripheral and spinal level induces intense analgesia by inhibition of excitatory transmission or via stimulation of descending antinociceptive pathway [22]. SA, a potent non-opioid selective KOR agonist, is the main active component of the plant *Salvia Divinorum*[23,24]. It exerts intense hallucinogenic effects, such as to lead to a kind of “out of the body” experience comparable to lysergic acid diethylamide effect [25]. SA has been demonstrated to be effective in some acute pain models [26-29] and we have recently demonstrated that it has an ultrapotent anti-inflammatory effect in LPS- and carrageenan-induced paw oedema [30].

In this study we have evaluated the effects of SA on formalin-induced pain symptoms. We have evaluated the possible anti-allodynic effect of daily SA treatment up to 7 days after subcutaneous formalin injection in mice. Moreover, we performed electrophysiological recordings of nociceptive neurons (NS), in order to investigate a possible neuronal activity changes induced by SA in formalin-injected mice. Finally, the involvement of glial and microglial cells and the levels of pro- and anti-inflammatory mediators in the SA-mediated effects at the spinal cord level was also investigated.

## Results

### SA repeated treatment reduces formalin-induced mechanical allodynia in KOR and CB1R sensitive manner

**Figure 1:**
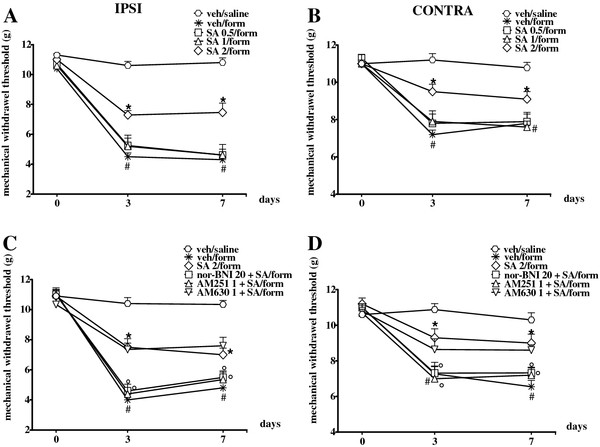
**Effect of SA (0.5, 1 and 2 mg/kg i.p.) repeated treatment on mechanical withdrawal threshold of the ipsi- (A, C) and contralateral (B, D) hind paw 3 and 7 days after formalin injection in mouse.** SA (2 mg/kg i.p.) was also administered in combination with nor-BNI (20 mg/kg i.p.), AM251 (1 mg/kg i.p.) or AM630 (1 mg/kg i.p.) (**C**, **D**). Data were expressed as mean ± SEM of mechanical withdrawal thresholds in grams. # indicate significant differences vs veh/saline-treated mice, * indicate significant differences vs vehicle treatment in formalin injected mice and ° indicate significant differences vs SA/formalin injected mice. P values <0.05 were considered statistically significant (one-way ANOVA followed by Student-Newman Keuls).

**Figure 2:**
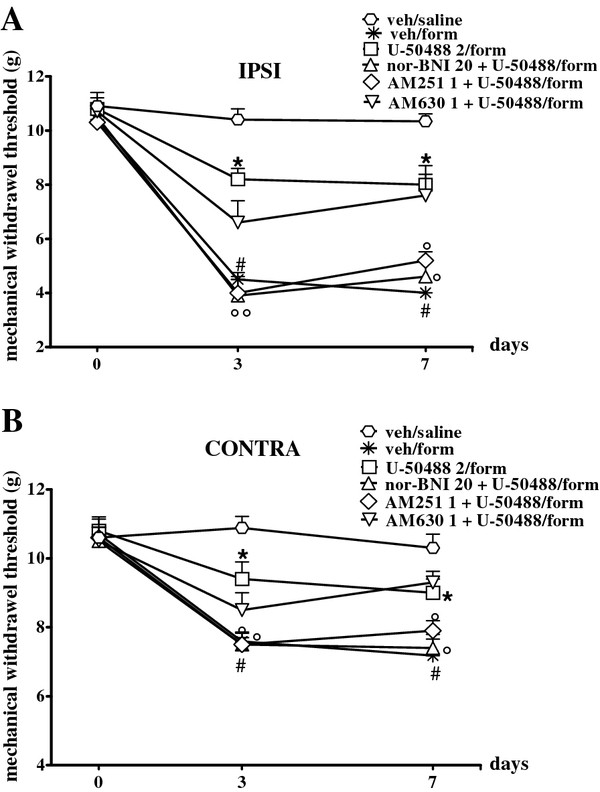
**Effects of U-50488 (2 mg/kg i.p.) repeated treatment alone or in combination with nor-BNI (20 mg/kg i.p.), AM251 (1 mg/kg i.p.) or AM630 (mg/kg i.p.) on mechanical withdrawal threshold of the the ipsi- (A) and contralateral (B) hind-paw 3 and 7 days after formalin injection in mouse.** Data were expressed as mean ± S.E.M of mechanical withdrawal thresholds in grams. # indicate significant differences vs veh/saline treated mice, * indicate significant differences vs vehicle treatment in formalin injected mice and ° indicate significant differences vs U-50488 /formalin injected mice. P values <0.05 were considered statistically significant (one-way ANOVA followed by Student-Newman Keuls).

Formalin injection on the dorsal surface of hind-paw induced an increase in paw volume and spontaneous pain behaviour. Accordingly with previous studies [14,17] we found a significant decrease of mechanical threshold in the injected and contralateral paw 3 and 7 days after formalin administration as compared to the saline-injected mice (Figure 1A and B). The subcutaneous injection of saline into the paw or intraperitoneal vehicle administration did not change the pain response as compare with naive mice (not shown). We found that SA (2 mg/kg, i.p.) daily treatment significantly reduced the formalin-induced mechanical allodynia at 3 and 7 days in both ipsi and contralateral paws. Lower doses of SA (0.5 and 1 mg/kg) did not exert any changes (Figure 1A and B). The SA anti-allodynic effect was prevented by the concurrent treatment with nor-BNI (20 mg/kg, i.p.), a KOR selective antagonist, and with AM251 (1 mg/kg i.p.), a CB1 receptor selective antagonist, but not with AM630 (1 mg/kg i.p.), a selective CB2 receptor antagonist (1 mg/kg i.p.) (Figure 1C and D). We also compared the effects of SA to those of U-50488, a well-established selective synthetic KOR agonist. Similarly to SA, U-50488 (2 mg/kg, i.p.) had anti-allodynic properties which were prevented by KOR and CB1R selective antagonists, but not by a CB2R selective antagonist (Figure 2A and B).

Moreover, we observed that chronic treatment with AM251 or nor-BNI, but not AM630, slightly increased the nociceptive response induced by formalin injection, even if the effect was not significant (not shown).

The dose of SA used (2 mg/kg i. p.) did not change the normal behaviours, neither induced sedation or motor impairment in saline or formalin-treated mice.

### Effect of SA on the NS neurons activity in formalin-treated mice

**Figure 3:**
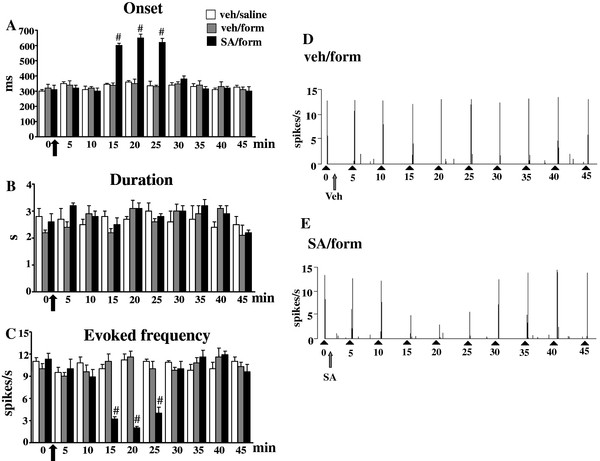
**Effects of spinal cord application of vehicle (0.05% DMSO in aCSF) or SA (50 μg/10 μl) on the onset (A), duration (B) and evoked frequency (C) of NS neurons in mice which received saline or formalin injection into the hind-paw.** Vehicle or SA were administered 3 days after saline or formalin as indicated by black arrows. Each point represents the mean ± S.E.M of 6–8 neurons of different treated groups of mice. # indicates significant differences vs veh/form. P values <0.05 were considered statistically significant (one-way ANOVA followed by Student-Newman Keuls). Representative ratemeters (**D** and **E**) show the responses to a noxious stimulation (von Frey filaments 97.8 mN/2 sec) of a single NS neuron both before and after vehicle or SA spinal application. Grey arrow indicates vehicle or SA application, while black triangle indicate the noxious stimulation on the mouse hind-paw.

**Figure 4:**
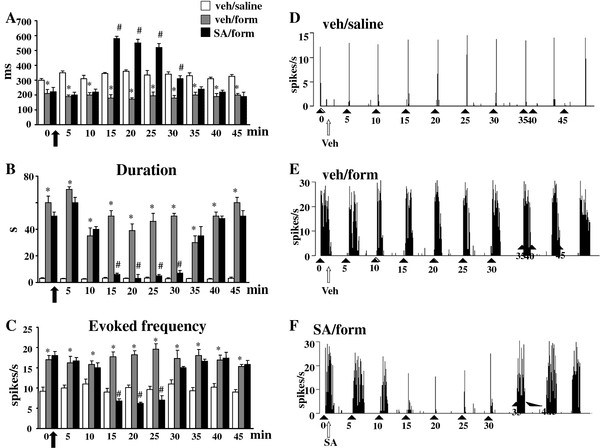
**Effects of spinal cord application of vehicle (0.05% DMSO in aCSF) or SA (50 μg/10 μl) on the onset (A), duration (B) and evoked frequency (C) of NS neurons in mice which received saline or formalin injection into the hind-paw.** Vehicle or SA were administered 7 days after saline or formalin peripheral injection as indicated by black arrows. Each point represents the mean ± S.E.M of 6–8 neurons of different treated groups of mice. * indicates statistically significant differences vs veh/saline and # indicates statistically significant differences *vs* veh/form. P values <0.05 were considered statistically significant (one-way ANOVA followed by Student-Newman Keuls). Representative ratemeters (**D**, **E** and **F**) show the responses to a noxious stimulation (von Frey filaments 97.8 mN/2 sec) of a single NS neuron both before and after vehicle or SA (50 μg/10 μl) spinal application. Grey arrow indicates vehicle or SA application, while black triangle indicate the noxious stimulation on the mouse hind-paw.

**Figure 5:**
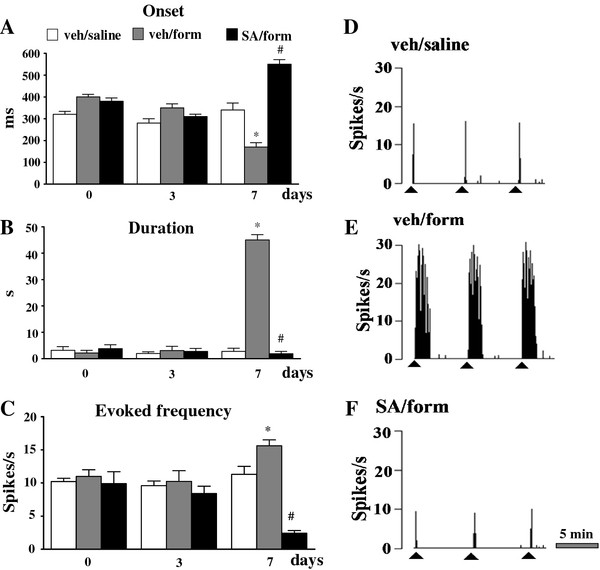
**Effects of vehicle (0.05% DMSO in aCSF) or SA (2 mg/kg, i.p.) repeated treatment on the onset (A), duration (B) and evoked frequency (C) of NS neurons 3 or 7 days after the injection of saline or formalin into the hind-paws.** Each point represents the mean ± S.E.M of 6–8 neurons of different treated groups of mice. * indicates statistically significant differences vs veh/saline and # indicates statistically significant differences versus veh/form. P values <0.05 were considered statistically significant (one-way ANOVA followed by Student-Newman Keuls). Ratemeters show the responses to a noxious stimulation (von Frey filaments 97.8 mN/2 sec) of a single representative NS neuron in mice receiving veh/saline (**D**), veh/form (**E**) or SA/form (**F**). Black triangles indicate the noxious stimulation on mouse hind-paw. Scale bar indicates 5 min intervals for ratemeter records.

The *in vivo* electrophysiological experiments were performed in order to investigate possible changes on NS neuron activity in formalin-induced inflammatory pain conditions with or without SA. The results are based on NS neurons (one cell recorded from each animal per treatment) at a depth of 0.7-1 mm from the surface of the spinal cord. This cell population was characterized by a mean rate of spontaneous firing of 0.015 ± 0.002 spikes/sec and only cells showing this pattern of basal firing were chosen for the experiment. Saline or formalin paw-injection did not change the spontaneous activity of NS neurons as shown in Figures 3, 4 and 5, while we observed significant changes in the evoked neuronal activity, measured by analyzing different parameters as onset, duration and frequency, 7 days after the formalin injection. We observed that formalin did not modify neuronal activity 3 days post-injection. SA application locally to the spinal cord, however, increased the onset and reduced the frequency of the evoked activity as compared to both mice receiving intra-paw formalin and vehicle and mice receiving intra-paw saline and vehicle (Figure 3 A and C). We found a significant increase in duration and frequency and a decrease in the onset of the evoked activity (39 ± 5 s, 19.6 ± 1.3 spikes/s, 172 ± 9 ms, respectively; P <0.05) 7 days after formalin paw-injection as compared to mice receiving intra-paw saline (Figure 4 A, B and C). Topical spinal cord application of SA (50 μg/10 μl) transiently reversed the formalin-induced changes in duration, frequency and the onset of the evoked cell activity (3 ± 0.3 s, 6.2 ± 0.3 spikes/s, 550 ±2 5 ms P <0.05). Repeated SA treatment, at the dose effective in alleviating mechanical allodynia (2 mg/kg, i.p.), completely normalized the induced-formalin changes 7 days after formalin injection. In particular, SA reduced the duration and frequency, while increased the onset of the evoked activity (1.9 ± 0.9 s, 2.4 ±0.4 spikes/s, 550 ± 21 ms respectively; P <0.05) of NS neurons in formalin-injected mice as compared to vehicle (0.05% DMSO in aCSF), while no significant changes were found in 3 days SA-treated mice (Figure 5 A, B and C). Representative ratemater records show the activity of a single NS neuron before and after a single vehicle or SA spinal cord topical application (Figure 3 D and E and Figure 4 D, E and F) and repeated treatment with vehicle or SA in mice receiving saline or formalin into the hind-paw (Figure 5 D, E and F).

### Changes in expression of cannabinoid and opioid receptors after formalin injection

**Figure 6:**
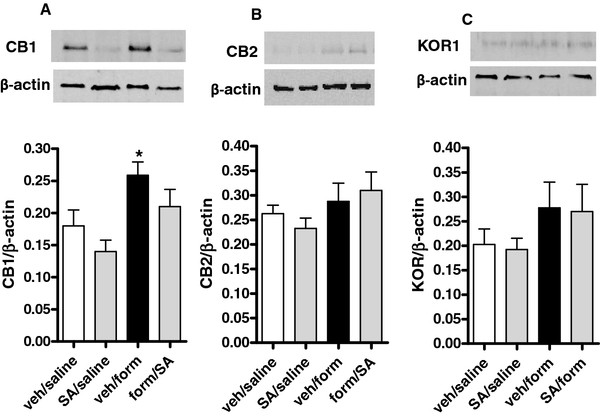
**Effect of vehicle or SA on CB1, CB2 and KOR expression in the dorsal horn of formalin-injected mice (A-C).** Data are expressed as mean ± S.E.M of 3 mice per group. * indicates statistically significant differences vs veh/saline 3 day after formalin (P <0.05, t-test).

Western blot analysis revealed a significant up-regulation of CB1 receptor (but not CB2 receptor or KOR) protein levels 3 days after formalin administration as compared with saline-treated mice (Figure 6). No significant changes have been revealed 7 days after formalin injection (not shown).

**Figure 7:**
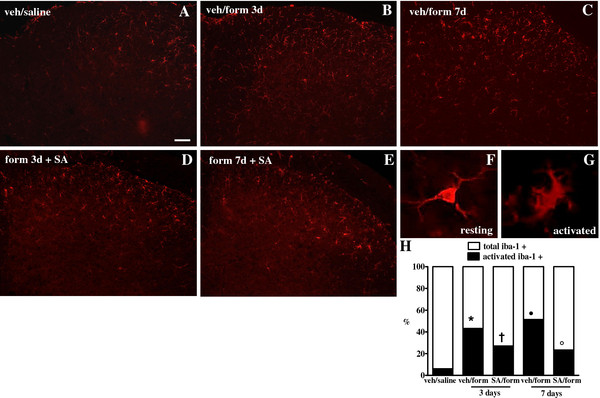
**Effect of vehicle or SA repeated treatment (2 mg/kg, i.p.) on spinal microglial cells in mice receiving saline or formalin into the hind-paws.** Iba-1 immunoreactivity (Iba-1-ir) is shown in the ipsilateral dorsal horn 3 (**B** and **D**) and 7 (**C** and **E**) days after saline or formalin (**A**). “**F**” and ‘**G**” represent examples of resting and activated microglia cell morphology, respectively. Quantitative analysis of percentage of activated microglial cells on the total cell number in L4-L6 spinal cord sections is shown in “**H**”. Data are expressed as mean ± S.E.M of 3 mice per group. * and **·** indicate statistically significant differences vs veh/saline. † indicates statistically significant differences vs veh/form 3 day after formalin and ° indicates statistically significant differences vs veh/form 7 days after formalin (P <0.05, one-way ANOVA, Tukey *post hoc*).

**Figure 8:**
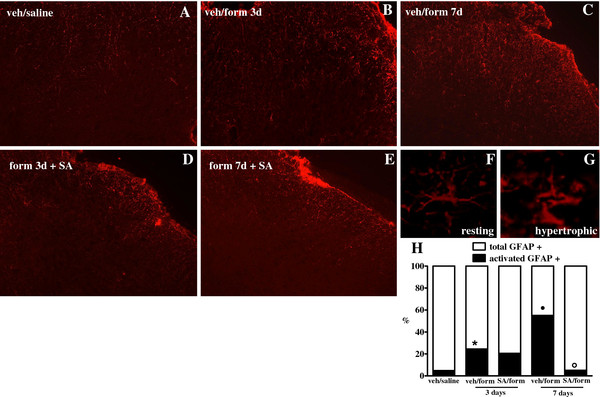
**Effect of SA repeated treatment (2 mg/kg, i.p.) on spinal astrocytes in mice receiving saline or formalin into the hind-paws.** GFAP immunoreactivity (GFAP-ir) is shown in the ipsilateral dorsal horn 3 (**B** and **C**) and 7 (**D** and **E**) days post-saline (**A**) or formalin. “**F**” and ‘**G**” represent examples of resting and activated astrocytes morphology, respectively. Quantitative analysis of percentage of hypertrophic astrocytes on the total cell number in L4-L6 spinal cord sections is shown in “**H**”. Data are expressed as mean ± S.E.M of 3 mice. * and **·** indicate statistically significant differences vs veh/saline. **°** indicates statistically significant differences vs veh/form 7 days after formalin injection, one-way ANOVA, Tukey *post hoc*).

SA reduces formalin-evoked glial and microglial activation at spinal cord level Immunohistochemical evaluations were performed in order to determine the occurrence of spinal microglia and astrocytes activation following intra-paw formalin injection. Morphological analysis of Iba-1 positive cells, accordingly to the criteria described by Hains and Waxman [31], revealed that formalin increased the number of activated microglial cells in the ipsilateral dorsal horn (L4–L6) 3 and 7 days post-injection as compared to saline-treated mice (Figure 7A, B and C). SA repeated treatment (2 mg/kg, i.p.) significantly reduced the number of morphologically activated microglia cells induced by formalin injection (Figure 7D and E). In addition, we observed that formalin increased the number of hypertrophic GFAP-labelled astrocytes 3 and even more 7 days post-injection as compared to saline-injected mice (Figure 8C). SA repeated treatment (2 mg/kg, i.p.) strongly abolished the formalin-induced reactive gliosis 7 days post-formalin injection as shown in Figure 8E. In a separate set of experiments, we have evaluated the involvement of CB1R and KOR in the SA-mediated effects on spinal microglia and astrocytes activation. We decided to perform these evaluations 7 days post formalin injection, which represent the peak of maximum cells activation. In particular, we found that AM251 treatment abolished the SA effect on the activation of both microglia and astrocytes, while a slight reduction of SA effects was found in nor-BNI-treated mice only in astrocytes activation (Additional file 1: Figure S1 and Additional file 2: Figure S2).

### SA modulates spinal IL-10 and i-NOS expression in formalin-injected mice

**Figure 9:**
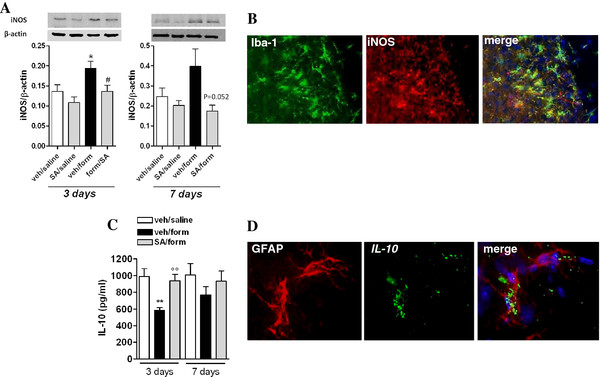
**Effect of SA (2 mg/kg, ip) on i-NOS expression (A) and IL-10 levels (C) in mice receiving saline or formalin into the hind paw (3 and 7 days).** Data are expressed as mean ± S.E.M (n = 3) in “**A**” * indicates significant differences vs veh/saline and # indicates significant differences vs veh/form (P <0.05, t-test). In “**C**” ** indicates significant differences vs veh/saline and ° indicates statistically significant differences vs veh/form (P <0.05, t-test). “**B**” represents iNOS expression up-regulation in the ipsilateral dorsal horn. Double labeling shows the iNOS expression in microglia. “**D**” represents IL-10 levels up-regulation in the ipsilateral dorsal horn. Double labeling shows the IL-10 level in astrocytes.

Formalin injection caused hyper-expression of i-NOS, an effect which was statistically significant 3, but not 7, days after its injection (Figure 9A). The increase in iNOS expression, which was found over-expressed in Iba-1 labelled microglial cells (Figure 9B), was normalised by SA (Figure 9A).

Similarly, IL-10 levels were significantly down-regulated 3, but not 7, days after formalin injection in the spinal cord (Figure 9C). The 3-days treatment with SA normalized IL-10 levels (Figure 9C). Moreover, by using an immunohistochemical approach, we were able to detect IL-10 in GFAP labelled astrocytes only in the spinal cord of SA treated mice (Figure 9D). Very weak staining was detectable in the saline or formalin-injected mice (not shown).

## Discussion

The main finding of this study is the anti-allodynic effect of SA, the main compound of *Salvia Divinorum*, in a formalin-induced chronic pain model in mouse. We have here coupled behavioural pharmacology with in vivo electrophysiology in order to: 1) clarify the long-lasting behavioural responses and NS neuronal activity induced by a single formalin injection 2) investigate the contribute of KOR/CB1 receptor manipulation in formalin-induced pain condition. In particular, we have shown that SA repeated treatment, reduces mechanical allodynia up to 7 days after formalin peripheral administration in mice. The local injection of formalin into the hind-paw of rodents has been considered mainly a model of persistent inflammatory pain, which induces a characteristic nocifensive biphasic response, oedema and inflammation [32]. We have previously demonstrated that a single administration of SA partially reduced the first phase of formalin-induced nociceptive behaviour, which is caused by direct activation of nociceptive sensory afferents, and it completely abolished the second one [30] which is due to the release of inflammatory mediators and it is also associated with central sensitization [33-35]. However, peripheral formalin injection also exerts long-term nerve injury [15,17,36,37]. Accordingly, we have shown that formalin leads to tactile allodynia ipsilaterally to the injection side spreading also to the contralateral hind-paw. SA was effective in reducing the formalin-induced ipsi- and contralateral allodynia up to 7 day treatment. Moreover, we found that SA effects were prevented by KOR and CB1R selective antagonists. Indeed, the involvement of CB1 receptor in SA-mediated effects has been already shown in other inflammatory animal models [30,38] and also in emotional behaviour studies in rodents [39]. How SA can reduce pain transmission by modulating CB receptors is presently unclear. SA shows very low or no affinity for CB1 and CB2 receptors, respectively, and it seems to be unable to modulate significantly endocannabinoid levels [38,40]. The finding that the effect of U-50488, the synthetic KOR agonist, was counteracted by nor-BNI and AM251 similarly to SA, suggests a possible functional KOR-CB1R interaction rather than an exclusive effect of the drug. Several findings have suggested that a functional cross-talk between cannabinoid and opioid systems exists [41-43], even if the mechanisms of such interaction are not still clear. A direct receptor–receptor interaction, such as heteromerization, as already suggested by Rios et al., [44] for CB1R and MOR opioid receptor, could represent one of possible mechanisms. Indeed, cannabinoids and opioids mutual interaction has been described with particular emphasis on the pivotal role of opioid receptors and peptides in cannabinoid-mediated analgesia [41,43] and importantly, such a functional interaction between the two systems seem to be altered during chronic pain [45]. In our model, we found a significantly increase of spinal CB1 receptor expression, while no changes have been detected for CB2R or KOR. The involvement of the CB2R, which is known to play an important role in chronic inflammatory process and also in reducing pain behaviour associated with neuropathic pain [13,46-48] has been also object of this study. The co-administration of SA with AM630, the selective CB2 receptor antagonist, ruled out the contribute of this receptor in SA-mediated effects.

Inflammation, tissue damage or nervous system injury result in the activation of immune and inflammatory cells, leading to consequent production of several mediators able to increase pain hypersensitivity. By using immunohistochemical approaches, we have found that formalin induces an increase of Iba-1 staining, a marker of microglia cells, in the dorsal horn of lumbar spinal cord ipsilaterally to the injection, as previously demonstrated [15,49]. In this study we also have observed an increase of hypertrophic GFAP positive cells 7 days post-injection in the same area. Astrocytes and microglia cells showed a typical morphological shape corresponding to the activated phenotype. The effectiveness of SA in alleviating mechanical allodynia matched with a reduction of the activated cells number and with the modulation of IL-10 and iNOS, two different markers involved in the inflammatory processes. In this study we found that, at time point evaluated, the IL-10 protein level was increased by SA treatment in terms of intracellular production compared to saline or formalin treated animals, as revealed by immunohistochemestry, while it was normalized in the released form, as revealed by ELISA. These data suggest that the mechanisms leading to the long-term nocifensive behaviour induced by formalin are correlated with a reorganization of the spinal cellular populations in which microglia and astrocytes activation play a significant role. In this context, the effectiveness of SA in reducing the activation of astrocytes and even more of microglia seems to be mainly mediated by CB1R rather than KOR. Indeed, we found that chronic treatment with AM251, but not nor-BNI, (see Additional file 1: Figure S1 and Additional file 2: Figure S2) reverted the effect of SA in reducing microglial activation. These data are consistent with the evidence showing that cannabinoids are more effective than opioids in alleviating neuropathic pain [50].

Importantly - and consistently with the behavioural data - electrophysiological experiments revealed that SA caused a strongly reduction of evoked activity of NS neurons in formalin-injected mice, suggesting that KOR/CB1R activation plays an important role in the transmission of noxious signals to the spinal cord under pathological conditions. Indeed, our findings are the first one showing the long-lasting effects of formalin injection on NS neurons activity. The decreased threshold of activation and the increased responsiveness to mechanical noxious stimuli of NS neurons found 7 days after formalin, suggest that a single peripheral formalin injection, beyond motor dysfunctions, induces a central sensitisation, similarly to a neuropathic pain condition induced by nerve injury. The finding that 7 days are needed to modulate spinal neuronal activity seems to be in contrast with allodynia development and with the lack of changes in spinal pro-inflammatory mediator level, observed already 3 days post-injection. However, this may be due to the formalin-induced intense inflammation with an immediate production of prostaglandins and accumulation of neutrophils and infiltrating mononuclear cells which could be alone responsible for the induction of allodynia. Indeed, a range of peripheral inflammatory stimuli, such as the peripheral injection of carageenan, mustard oil and complete Freund’s adjuvant, have been shown to induce a robust allodynia and hyperalgesia in adult animals [51,52]. In our model, the initial microglial activation and/or the release of pro-inflammatory cytokines appears to be unable to sensitize dorsal horn sensory neurons. It would be reasonable to hypothesize that microglia and astrocytes can participate together in neuronal sensitization, not only by releasing cytokines and various mediators, but also more directly via release of glutamate and/or by evoked-changes in synaptic ion currents [6]. The evidence of the crucial role of astrocytes in the maintenance of mechanical allodynia in chronic pain and our finding that astroglial activation does not occur until to the 7^th^ day after formalin injection, supports their involvement in the establishment of central sensitization. Moreover, some brain areas of the endogenous antinociceptive pathway, i.e. periaqueductal gray (PAG)-rostral ventromedial medulla (RVM) axis, could be activated in pathophysiological conditions associated with persistent pain, so reducing neuronal sensitization at spinal level. In fact, previous findings showing an increased endocannabinoid release within the dorsal and lateral PAG following a single formalin injection [53], suggest a possible activation of CB1 receptors in such areas in inflammatory conditions, which could be responsible for the inhibition of the spinal NS neurons over-excitability.

## Conclusion

In conclusion, this findings show that SA is effective in reducing formalin-induced mechanical allodynia with a mechanism involving KOR and CB1. Moreover, SA reduced spinal neuron hyperexcitability and the glial contribute in the establishment of some critical phenotypical changes in the spinal cord associated with chronic pain development. SA could provide a novel lead compound for developing anti-inflammatory/allodynic agents.

## Methods

### Animals and treatments

Male ICR (CD-1) mice (30–35 g) were housed 3 per cage under controlled illumination (12:12 h light:dark cycle; light on 06.00 h) and environmental conditions (room temperature 20-22°C, humidity 55-60%) for at least 1 week before the commencement of experiments. Mouse chow and tap water were available ad libitum. The experimental procedures were approved by the Animal Ethics Committee of the Second University of Naples. Animal care was in compliance with the IASP and European Community (E.C. L358/1 18/12/86) guidelines on the use and protection of animals in experimental research. All efforts were made to minimize animal suffering and to reduce the number of animals used. Groups of 6–8 mice for behavioral and electrophysiological experiments and groups of 3 mice for ex vivo studies received subcutaneous injection of 30 μl of saline (0.9% NaCl) or formalin (1.25%) into the dorsal surface of the right hind paw. Repeated treatments (i.p.) were performed ones a day and all the evaluations have been carried out before each injection. The doses used were in accord with our previous data [30].

For behavioral test, molecular evaluations and immunohistochemistry mice receiving saline or formalin into the hind-paw have been repeated (3 or 7 days) treated as follows:

1) vehicle (0.5% DMSO in 0.9% NaCl, i.p.) or SA (0.5, 1 and 2 mg/kg, i.p.).

2) SA (2 mg/kg i.p.), U-50488 (2 mg/kg i.p.) alone or in combination with nor-BNI (20 mg/kg, i.p.), AM251 (1 mg/kg, i.p.) and AM630 (1 mg/kg, i.p.).

For the electrophysiological recordings groups of 6–8 mice received subcutaneous injection of saline or formalin into the dorsal surface of the right hind paw and were treated as follows:

1) spinal topic application of vehicle (DMSO/aCSF, 0.05%, v/v) or SA (50 μg/10 μl).

2) repeated treatment (3 or 7 days) with vehicle (0.5% DMSO in 0.9% NaCl, i.p.) or SA (2 mg/kg i.p.).

### Nociceptive behavior

Mechanical allodynia was measured by using the Dynamic Plantar Aesthesiometer (Ugo Basile, Varese, Italy). Mice were allowed to move freely in one of the two compartments of the enclosure positioned on the metal grid surface. A mechanical stimulus was delivered to the plantar surface of the hind-paw of the mouse through the metal grid by an automated steel filament exerting an increasing force of 0–30 grams in 10 seconds. The force inducing paw withdrawal was recorded to the nearest 0.1 g. Nociceptive responses for mechanical sensitivity (mechanical withdrawal threshold) were measured in grams. Baseline thresholds were determined 6 days before starting with the treatments. The observer was blind to the treatments.

### Electrophysiological recordings

On the day of electrophysiological recording experiments, mice were initially anaesthetized with sodium pentobarbital (50 mg/kg, i.p.). After tracheal cannulation, a catheter was placed into the right external jugular vein, to allow continuous infusion of propofol (5–10 mg/kg/h, i.v.) and spinal cord segments L4-L6 were exposed by laminectomy, medially near the dorsal root entry zone up to a depth of 1000 μm. An elliptic rubber ring (about 3 × 5 mm) was tightly sealed with silicone gel onto the surface of the cord. This ring formed a trough with about 50 μl capacity over the spinal segments used for topical spinal drug application and to gain access to spinal neurons. Animals were then secured in a stereotaxic apparatus (David Kopf Instruments, Tujunga, CA, USA) supported by clamps attached to the vertebral processes on either side of the exposure site. Body temperature was maintained at 37°C with a temperature-controlled heating pad [54,55]. A glass-insulated tungsten filament electrode (3–5 MΩ) (FHC Frederick Haer & Co., ME, USA) was used to record single unit extracellular activity of dorsal horn NS neurons. NS neurons were defined as those neurons responding only to high-intensity (noxious) stimulation [54] for saline or formalin-injected animals, each neuron was characterized by giving a mechanical stimulation to the injected paw by von Frey filament with a bending force of 97.8 mN (noxious stimulation) for 2 s with it slightly buckled to confirm NS response patterns. Only neurons that specifically responded to noxious hind paw stimulation, without responding to stimulation of the surrounding skin/tissue, were considered for recordings. The recorded signals were amplified and displayed on a digital storage oscilloscope to ensure that the unit under study was unambiguously discriminated throughout the experiment. Signals were also fed into a window discriminator, whose output was processed by an interface CED 1401 (Cambridge Electronic Design Ltd., UK) connected to a Pentium III PC. Spike2 software (CED, version 4) was used to create peristimulus rate histograms on-line and to store and analyze digital records of single unit activity off-line. Configuration, shape, and height of the recorded action potentials were monitored and recorded continuously using a window discriminator and Spike2 software for on-line and off-line analysis. This study only included neurons whose spike configuration remained constant and could clearly be discriminated from activity in the background throughout the experiment, indicating that the activity from one neuron only and from the same neuron was measured. Spontaneous and evoked neuronal activity was recorded in different groups of animals after repeated vehicle or drug administration and local vehicle or drug application. The neuronal activity was expressed as spikes/sec (Hz) and only one neuron was recorded in each mouse. At the end of the experiment, each animal was killed with a lethal dose of pentobarbital.

### Spinal cord immunohistochemistry

Under pentobarbital anaesthesia (50 mg/kg, i.p.), animals were transcardially perfused with saline solution followed by 4% paraformaldehyde in 0.1 M phosphate buffer. The lumbar spinal cord was excised, post fixed for 3 h in the perfusion fixative, cryoprotected for 72 h in 30% sucrose in 0.1 M phosphate buffer and frozen in O.C.T. embedding compound. Transverse sections (20 μm) were cut using a cryostat and thaw-mounted onto glass slides. Slides were incubated overnight with primary antibody solutions for the microglial cell marker Iba-1 (rabbit anti-ionized calcium binding adapter molecule-1; 1:1000; Wako Chemicals, Germany), GFAP (rabbit poly-clonal anti-glial fibrillar acidic protein, 1:1000; Dako Cytomation, Denmark) IL-10 (goat anti-interleukin 10, 1:100; Santa Cruz, USA) or iNOs (mouse anti-iNOs BD Biosciences Pharmigen). A possible unspecific labeling of mouse secondary antibody has been detected by using secondary antibody alone. Following incubation sections were washed and incubated for 2 hrs with secondary antibody solution. Slides were washed, cover-slipped with Vectashield mounting medium (Vector Laboratories, USA) and visualized under a Leica fluorescence microscope.

### Quantitative image analysis

The number of profiles positive for Iba-1, GFAP and IL-10 were determined within a box measuring 10^4^ μm^2^ in the lateral, central and medial areas of the dorsal horn spinal cord sections, at both ipsilateral or contralateral sides. Eight L5 spinal sections were assessed from each of three animals per group, and a mean value obtained by combining values from lateral, central and medial areas of dorsal horn. To avoid cell over-counting, only DAPI-counterstained cells were considered as positive profiles. Resting and activated microglia were classified based on the following criteria: resting microglia displayed small somata bearing long, thin and ramified processes whereas morphologically activated microglia exhibited marked cellular hypertrophy and retraction of processes such that the process length was less than the diameter of the soma size. Cells were sampled only if the nucleus was visible within the plane of section and if cell profiles exhibited distinctly delineated borders. For astrocytes the hypertrophic cells have been identified as activated compared to the thin cells presenting long processes.

### Western blot analysis

Spinal cords were collected from each animal 3 and 7 days after formalin or saline injection, and they were homogenized on ice in lysis buffer containing 1x PBS, 1% Nonidet-P40, 0.5% sodium deoxycholate, 0.1% SDS, 1 mM PMSF, 1 mM Na_3_VO_4_ and complete protease inhibitor cocktail (Roche Diagnostics, Mannheim, Germany). Tissue lysates were centrifuged at 16.200 g for 15 min at 4°C, and the supernatants were used for protein determination and stored at −80°C until use.

Western blot analysis were performed *ex vivo* on spinal cord lysates of animals treated or not with formalin (alone or with SA 2 mg/kg) for 3 and 7 days, to investigate the expression of inducible nitric oxide synthase (iNOS). Protein lysates (70 μg) were separated on SDS-polyacrylamide gels, and membranes were incubated with anti-iNOS (BD Biosciences from Becton Dickinson, Buccinasco, Italy) and anti-β-actin (Sigma, Milan, Italy). Signals were visualized using ImageQuant 400 equipped with Quantity One Software 4.6.3 (GE Healthcare, Milan, Italy).

### Interleukin-10 level measurements

Interleukin-10 (IL-10) levels were measured on spinal cord lysates obtained from animals treated or not with formalin (alone or with SA 2 mg/kg) for 3 and 7 days as reported above, using a commercial ELISA kit (Boster Immunoleader from Tema Ricerca, Bologna, Italy) according to the manufacturer’s instructions.

### Chemicals

Salvinorin A (purity: 99% by HPLC) was isolated from *S. divinorum* leaves. 3,4-Dichloro-N-methyl-N-[2-(1-pyrrolidinyl)cyclohexyl]benzeneacetamide hydrochloride (U-50488) was purchased from Sigma (Milan, Italy); 17,17'-(Dicyclopropylmethyl)-6,6',7,7'-6,6'-imino- 7,7'-binorphinan-3,4',14,14'-tetrol dihydrochloride (nor-BNI), 1-(2,4-dichlorophenyl)-5-(4-iodophenyl)-4-methyl-N-piperidin-1-ylpyrazole-3-carboxamide (AM251) and 6-Iodo-2-methyl-1-[2-(4-morpholinyl)ethyl]-1 H-indol-3-yl](4-methoxyphenyl)methanone (AM630) were purchased from Tocris Cookson (Northpoint, UK). All reagents for western blot analysis were obtained from Sigma (Milan, Italy), Bio-Rad Laboratories (Milan, Italy) and Microglass Heim (Naples, Italy). All drugs used (i.e. SA, U-50488, nor-BNI, AM251 and AM630) were dissolved in 0.5% v/v DMSO in saline or aCFS, depending on the administration route.

### Data analysis

Behavioural and electrophysiological data were expressed as mean ± S.E.M. (n = 6–8) and analyzed by using the one-way analysis of variance (ANOVA) for repeated measures followed by the Student-Newman Keuls for multiple comparisons to determine statistical significance between different treated groups of mice. Immunohistochemical data were expressed as mean ± S.E.M (n = 3) and analyzed by using the one-way analysis of variance (ANOVA) for repeated measures followed by Tukey post hoc. Biomolecular analysis and protein quantifications were expressed as mean ± S.E.M (n = 3) and analyzed by t-test. P <0.05 was considered as significant.

## Competing interests

The authors declare that they have no competing interests.

## Authors’ contributions

FG conceived of the study and drafted the manuscript. SM and VdN participated in the design and coordination of the research. MDC and IM performed the behavioural evaluations. LL performed the immunoistochemistry. LG and SB carried out the electrophysiological recording. GA and RC carried out the bio-molecular evaluations. EP and AI helped to draft the manuscript and performed the statistical analysis. JKZ provided the Salvinorin A. All authors read and approved the final manuscript.

## Supplementary Material

Additional file 1 **Figure S1.** Effect of vehicle or SA repeated treatment (2 mg/kg, i.p.), alone or in presence of nor-BNI (20 mg/kg, i.p.), or AM251 (1 mg/kg, i.p.) on spinal microglial cells in mice receiving formalin into the hind-paws. Iba-1 immunoreactivity (Iba-1-ir) is shown in the ipsilateral dorsal horn 7 days after formalin (A-D). Quantitative analysis of percentage of activated microglial cells on the total cell number in L4-L6 spinal cord sections is shown in “E”. Data are expressed as mean ± S.E.M of 3 mice per group. * and ○ indicate statistically significant differences vs veh/form and SA/form, respectively. (P <0.05, one-way ANOVA, Tukey *post hoc*). (PPT 390 kb) Click here for file

Additional file 2 **Figure S2.** Effect of vehicle or SA repeated treatment (2 mg/kg, i.p.), alone or in presence of nor-BNI (20 mg/kg, i.p.), or AM251 (1 mg/kg, i.p.) on spinal astrocytes in mice receiving formalin into the hind-paws. GFAP immunoreactivity (GFAP-ir) is shown in the ipsilateral dorsal horn 7 days after formalin (A-D). Quantitative analysis of percentage of activated astrocytes on the total cell number in L4-L6 spinal cord sections is shown in “E”. Data are expressed as mean ± S.E.M of 3 mice per group. * and ○ indicate statistically significant differences vs veh/form and SA/form, respectively. (P <0.05, one-way ANOVA, Tukey *post hoc*). (PPT 303 kb) Click here for file
