# Supplementary figures and images for: Salvinorin A reduces mechanical allodynia and spinal neuronal hyperexcitability induced by peripheral formalin injection
Source: Mol Pain. 2012 Aug 23;8:60. doi: 10.1186/1744-8069-8-60 (PMC3522567; doi:10.1186/1744-8069-8-60)

## Slide 1
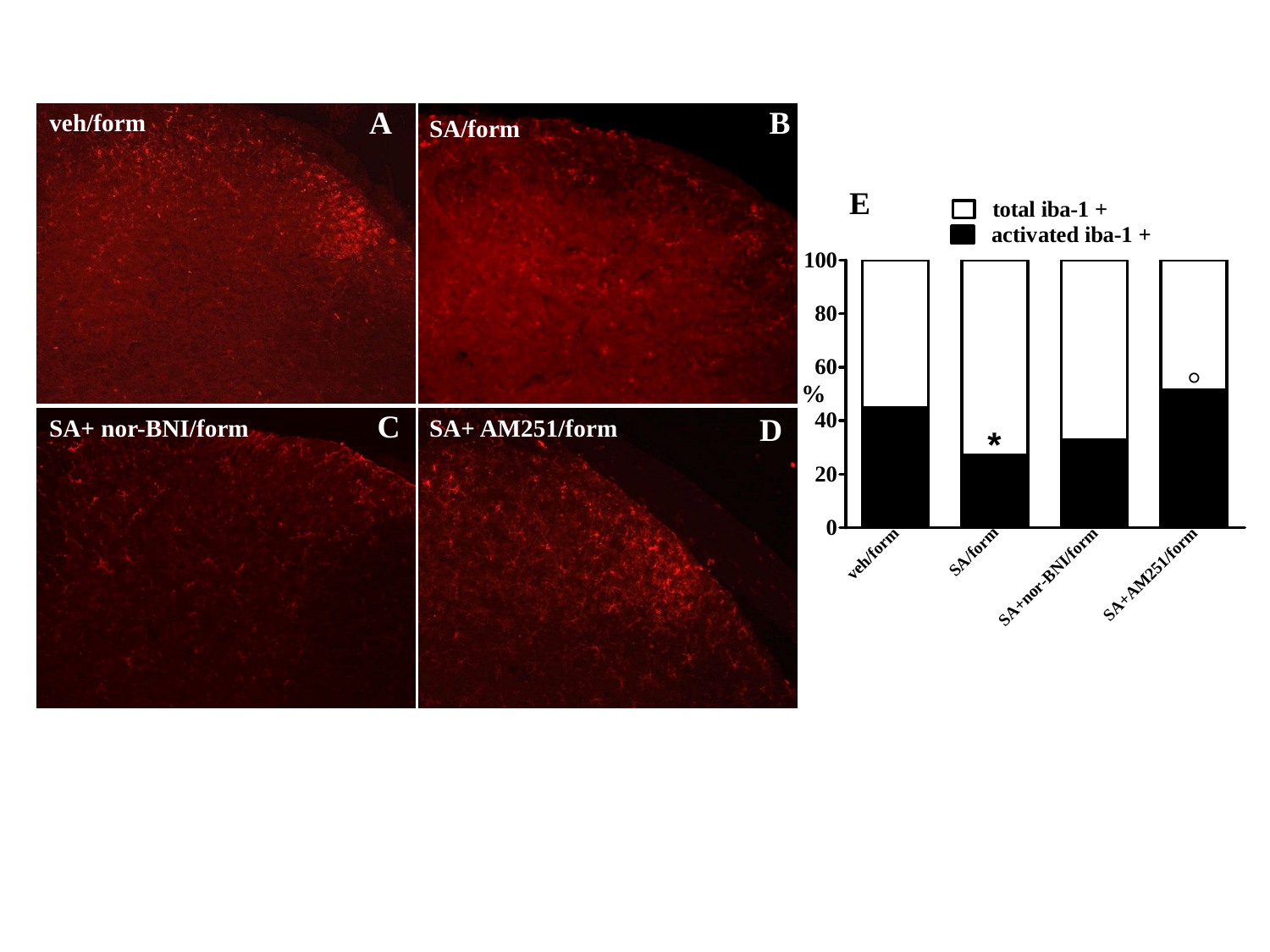

A
B
E
◦
%
D
*
veh/form
SA/form
C
SA+ nor-BNI/form
SA+ AM251/form

Supplement: Additional file 1 — Figure S1. Effect of vehicle or SA repeated treatment (2 mg/kg, i.p.), alone or in presence of nor-BNI (20 mg/kg, i.p.), or AM251 (1 mg/kg, i.p.) on spinal microglial cells in mice receiving formalin into the hind-paws. Iba-1 immunoreactivity (Iba-1-ir) is shown in the ipsilateral dorsal horn 7 days after formalin (A-D). Quantitative analysis of percentage of activated microglial cells on the total cell number in L4-L6 spinal cord sections is shown in “E”. Data are expressed as mean ± S.E.M of 3 mice per group. * and ○ indicate statistically significant differences vs veh/form and SA/form, respectively. (P <0.05, one-way ANOVA, Tukey post hoc). (PPT 390 kb) [file 1744-8069-8-60-S1.ppt]

## Slide 1
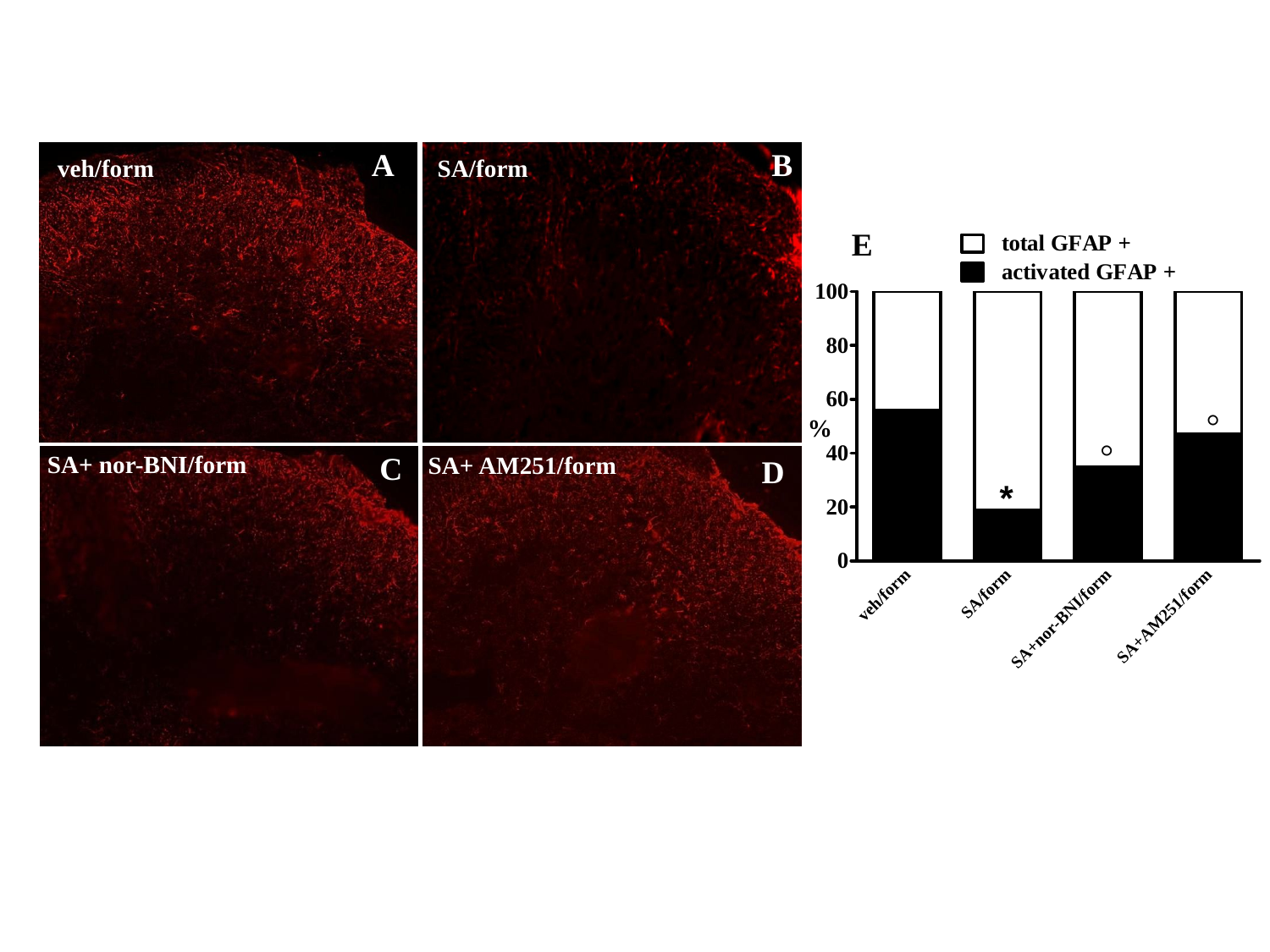

A
B
veh/form
SA/form
E
◦
%
◦
C
SA+ nor-BNI/form
SA+ AM251/form
D
*

Supplement: Additional file 2 — Figure S2. Effect of vehicle or SA repeated treatment (2 mg/kg, i.p.), alone or in presence of nor-BNI (20 mg/kg, i.p.), or AM251 (1 mg/kg, i.p.) on spinal astrocytes in mice receiving formalin into the hind-paws. GFAP immunoreactivity (GFAP-ir) is shown in the ipsilateral dorsal horn 7 days after formalin (A-D). Quantitative analysis of percentage of activated astrocytes on the total cell number in L4-L6 spinal cord sections is shown in “E”. Data are expressed as mean ± S.E.M of 3 mice per group. * and ○ indicate statistically significant differences vs veh/form and SA/form, respectively. (P <0.05, one-way ANOVA, Tukey post hoc). (PPT 303 kb) [file 1744-8069-8-60-S2.ppt]
